# Adipose mesenchymal stem cell-derived soluble factors, produced under hypoxic condition, efficiently support in vivo angiogenesis

**DOI:** 10.1038/s41420-023-01464-4

**Published:** 2023-05-23

**Authors:** Ludovica Barone, Maria Teresa Palano, Matteo Gallazzi, Martina Cucchiara, Federica Rossi, Marina Borgese, Mario Raspanti, Piero Antonio Zecca, Lorenzo Mortara, Roberto Papait, Giovanni Bernardini, Luigi Valdatta, Antonino Bruno, Rosalba Gornati

**Affiliations:** 1 Laboratory of Cell Biology, Department of Biotechnology and Life Sciences, University of Insubria, 21100 Varese, Italy; 2 Laboratory of Innate Immunity, Unit of Molecular Pathology, Biochemistry and Immunology, Istituto di Ricovero e Cura a Carattere Scientifico (IRCCS) MultiMedica, 20138 Milan, Italy; 3 Immunology and General Pathology Laboratory, Department of Biotechnology and Life Sciences, University of Insubria, 21100 Varese, Italy; 4 Department of Medicine and Surgery, University of Insubria, 21100 Varese, Italy; 5 Unit of Plastic and Reconstructive Surgery, Department of Biotechnology and Life Sciences, University of Insubria, 21100 Varese, Italy

**Keywords:** Mesenchymal stem cells, Stem cells

## Abstract

Tissue regeneration or healing both require efficient vascularization within a tissue-damaged area. Based on this concept, a remarkable number of strategies, aimed at developing new tools to support re-vascularization of damaged tissue have emerged. Among the strategies proposed, the use of pro-angiogenic soluble factors, as a cell-free tool, appears as a promising approach, able to overcome the issues concerning the direct use of cells for regenerative medicine therapy. Here, we compared the effectiveness of adipose mesenchymal stem cells (ASCs), use as cell suspension, ASC protein extract or ASC-conditioned-medium (i.e., soluble factors), combined with collagenic scaffold, in supporting in vivo angiogenesis. We also tested the capability of hypoxia in increasing the efficiency of ASC to promote angiogenesis, via soluble factors, both in vivo and in vitro. In vivo studies were performed using the Integra® Flowable Wound Matrix, and the Ultimatrix in sponge assay. Flow cytometry was used to characterize the scaffold- and sponge-infiltrating cells. Real-time PCR was used to evaluate the expression of pro-angiogenic factors by stimulating Human Umbilical-Vein Endothelial Cells with ASC-conditioned media, obtained in hypoxic and normoxic conditions. We found that, in vivo, ACS-conditioned media can support angiogenesis similar to ASCs and ASC protein extract. Also, we observed that hypoxia increases the pro-angiogenic activities of ASC-conditioned media, compared to normoxia, by generating a secretome enriched in pro-angiogenic soluble factors, with bFGF, Adiponectine, ENA78, GRO, GRO-a, and ICAM1-3, as most regulated factors. Finally, ASC-conditioned media, produced in hypoxic condition, induce the expression of pro-angiogenic molecules in HUVECs. Our results provide evidence that ASC-conditioned-medium can be proposed as a cell-free preparation able to support angiogenesis, thus providing a relevant tool to overcome the issues and restrictions associated with the use of cells.

## Introduction

Angiogenesis represents a crucial step in several physiological events, like embryo development and tissue repair [1–3]. Angiogenesis can be promoted by endothelial and non-endothelial cells [4–7]. Several stromal and immune cells undergo the angiogenic switch due to their cell plasticity and the increased demand for nutrients and oxygen by damaged tissues [6–11]. In this scenario, macrophages represent the classical prototype, being able, through the M2-like polarization, to actively support angiogenesis during tissue repair [12–15].

Angiogenesis and extracellular matrix (ECM) remodeling are two closely interacting events in tissue repair. ECM is a three-dimensional network of macromolecules, produced by resident cells, that affect cell signaling [16, 17]. Following a trauma, stromal cells can generate a unique microenvironment that remodels the surrounding matrix to support suitable cellular processes [18, 19]. In the last decade increased attention has been addressed to the formulation of natural and synthetic 3D matrices that, by interacting with cell surface receptors, or through paracrine pathways are able to modulate specific responses of homing cells, towards the reestablishment of normal tissue architecture [20–24].

Guiding the development and tissue integration of vascular networks is pivotal for regenerative medicine, as it plays a crucial role in the construction of engineered bio-scaffold, ensuring their successful grafting. Here, a major challenge, apart from biocompatibility, is the capability of bio-scaffold to efficiently induce cell recruiting while maintaining their functionality.

As an optimal vascularization is necessary to promote tissue regeneration of the damaged area, a remarkable number of strategies, involving the use of Mesenchymal Stem Cells (MSCs), substrate materials, and biochemical cues have emerged. MSCs represent the ideal tool, since they are present in almost all tissues, can be easily isolated and maintained, can differentiate into almost any end-stage lineage cells, and can be seeded in specific scaffolds. Moreover, several MSC-based randomized clinical trials for ischemic heart disease therapy are being registered and completed [25–32].

However, considering limitations and restrictions on the use of MSCs, including cell mortality rate, anti-angiogenic activities [33–35] and immunosuppressive functions [36–38], a cell-free therapy approach is well regarded. In this context, of particular importance is the cell secretome, an assortment of growth factors (GF) and cytokines that guide proliferation, adhesion, and differentiation of stem and progenitor cells towards the formation of functional vascular networks. The ancestor of cell secretome is the Platelet-Rich-Plasma (PRP), also known as autologous conditioned plasma, which consists of various growth factors (Platelet-derived (PDGF), vascular endothelial (VEGF), basic fibroblast (bFGF), Hepatocyte (HGF), and Transforming Growth Factor (TGF) secreted by platelets [39, 40], and recent clinical trials demonstrated its beneficial use in the healing process [41, 42]. These encouraging results have pushed the research toward the study of cell secretome which also includes the extracellular fraction [43].

In the case of new vascularization strategies aimed at tissue regeneration, it is essential to localize the action area, and in this regard, by in vivo experiments on nude mice, it has been demonstrated that a collagen-nanostructured scaffold, loaded with MSC secretome, exerts a synergic positive effect on angiogenesis [23]. Indeed, the use of MSC secretome, here referred as MSC-conditioned media (MSC-CMs), has been considered as “the new paradigm towards cell-free therapeutic mode for regenerative medicine” [44].

Here, we explored and compared the pro-angiogenic activities of adipose-derived mesenchymal stem cells (ASCs) as cell component, ASC protein extract and ASC-CMs, generated under normoxic and hypoxic condition, in supporting angiogenesis, in vitro and in vivo.

Integra® Flowable Wound Matrix (FWM), loaded with ASCs, ASC protein extract, or ASC-CMs and grafted for 28 days in athymic BALB/c nude mice, share comparable capability to promote cell infiltration and capillary growth in vivo. Similar results were observed using the Utimatrix sponge assay in vivo, where we found increased vascularization induced by ASC-CMs. Finally, by characterizing the ASC-CMs in normoxic and hypoxic conditions, we found an enrichment in different pro-angiogenic factors (as bFGF, Adiponectine, C-X-C motif chemokines, intracellular adhesion molecules 1 and 3 (ICAM1-3), interleukines) that further explain the transcriptional activation of human umbilical endothelial vein cells (HUVEC), exposed to ASC-CMs.

## Results

### Pro-angiogenic activities of MSCs and their products, in in vivo FWM scaffolds

After 28 days from grafting, SEM observation (Supplementary Fig. 1) indicates that the collagen ultrastructure of FWM alone (Supplementary Fig. 1A) was maintained and comparable to that reported by the dealer. SEM images of FWM associated with cells supported the assumption that the collagen fibrils were synthetized by fibroblasts that colonized the scaffold (Supplementary Fig. 1B and C).

**Fig. 1:**
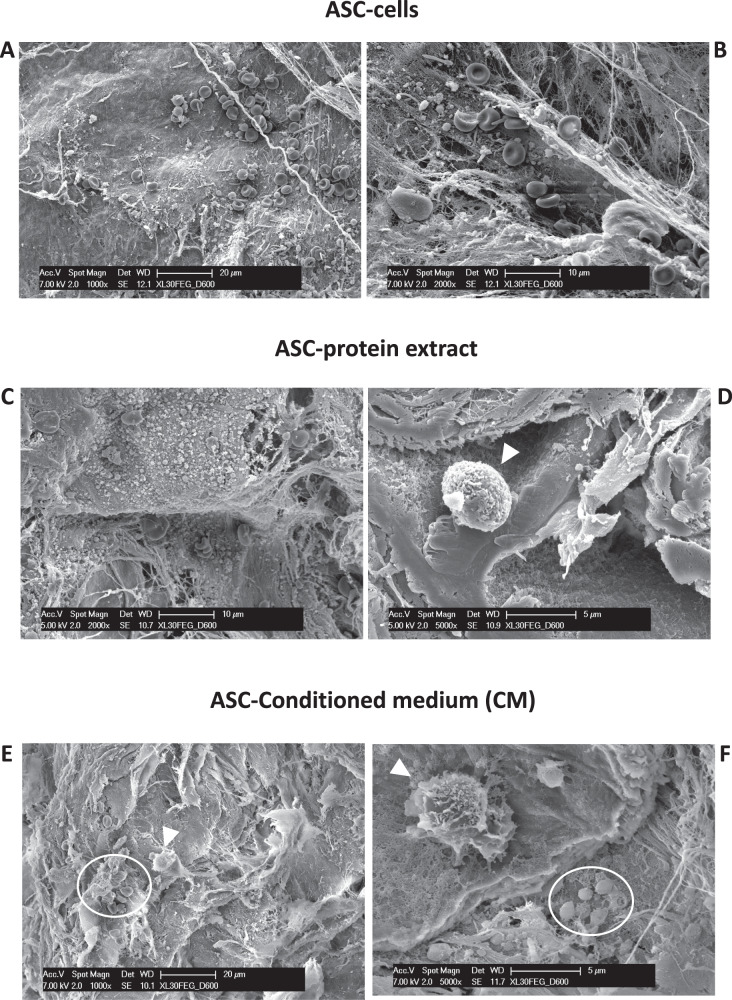
Representative SEM images of the scaffold FWM, associated with the designed formulations after grafting. ASCs (**A**, **B**), ASC-protein extract (**C**, **D**), ASC-conditioned medium (**E**, **F**). Scale bars are indicated in the pictures. **A**–**C** Beside the collagen fibrils is evident the infiltration of erythrocytes, clear indication of the presence of blood vessels within the scaffold. Magnification of picture **A**, ×1000; Magnification of picture **B**, ×2000; Magnification of picture **C**, ×2000. **D** Leucocytes, probably macrophages, were also present (arrowhead). Picture magnification ×5000. **E** Erythrocytes (circle) and a leucocyte, probably macrophage (arrowhead), are here evident. **F** In this magnification of the picture **E**, beside a leucocyte, platelets (circle) are noted, corroborating the evidence regarding the vascularization occurred inside the scaffold.

**Fig. 2:**
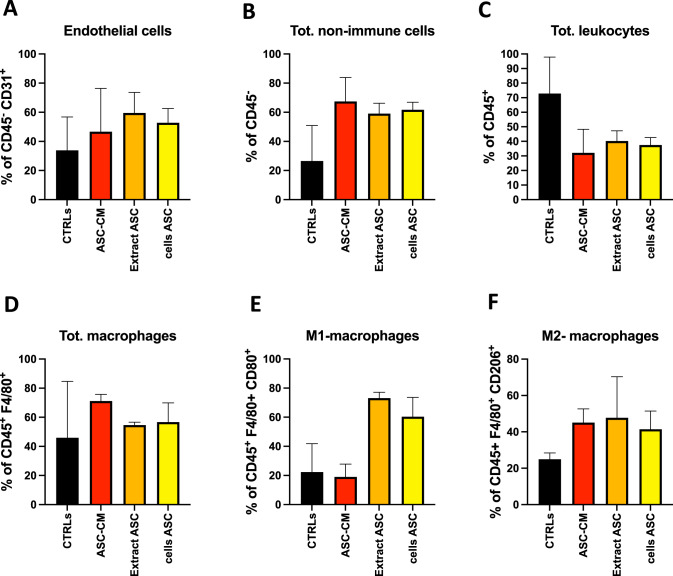
FACS analysis for cell infiltration in Integra scaffold. Cell population infiltrating the Integra scaffold were evaluated by flow cytometry and identified as CD45^-^CD31^+^ endothelial cells (**A**), CD45^-^ non-immune cells (**B**), CD45^+^ total leukocytes (**C**), CD45^+^F4/80^+^ total macrophages (**D**), CD45^+^F4/80^+^CD80^+^ M1-like macrophages (**E**), CD45^+^F4/80^+^CD206^+^ M2-like macrophages (**F**). Results are shown as mean ± SEM, ANOVA. CTRLs (empty scaffold), CM-ASC (conditioned media of ASCs), Extract ASC (protein extract), cells ASC (cells ASC). Each bar shows values from 3 mice per experimental condition (*n* = 12).

In the formulations FWM-ASCs, FWM-cell protein extract, and FWM-CMs, the massive presence of erythrocytes (Fig. 1A, C, E), and platelets (Fig. 1F, within the circle), corroborated our previous results regarding the vascularization occurred inside the scaffold [23]. Macrophages were also present (Fig. 1D–F, arrow heads), as confirmed by FACS analysis (Fig. 2). In all the grafted scaffolds, necrotic areas, cyst formation or fibrosis were not observed.

We better characterized the cell populations found in the recovered FWM scaffolds, by flow cytometry. FACS analysis of the single cell suspension, obtained from the enzymatically digested FWM scaffolds, revealed the efficient infiltration of different cell populations, that includes CD45^-^ stromal cells, CD45^-^CD31^+^ endothelial cells, CD45^+^ leukocytes and F4/80^+^ macrophages (Fig. 2A–D). Extending the FACS characterization to the F4/80^+^ cell infiltrate, we found enrichment of pro-inflammatory CD80^+^ M1-macrophages (Fig. 2E) in those scaffolds associated with ASC protein extract and ASCs, while the frequency of pro-angiogenic CD206^+^ M2-like macrophages (Fig. 2F) was comparable among scaffolds associated with ASC-conditioned medium (ASC-CM), ASC-protein extract and ASCs.

### Effect of hypoxia on pro-angiogenic functions of ASCs in vivo

**Fig. 3:**
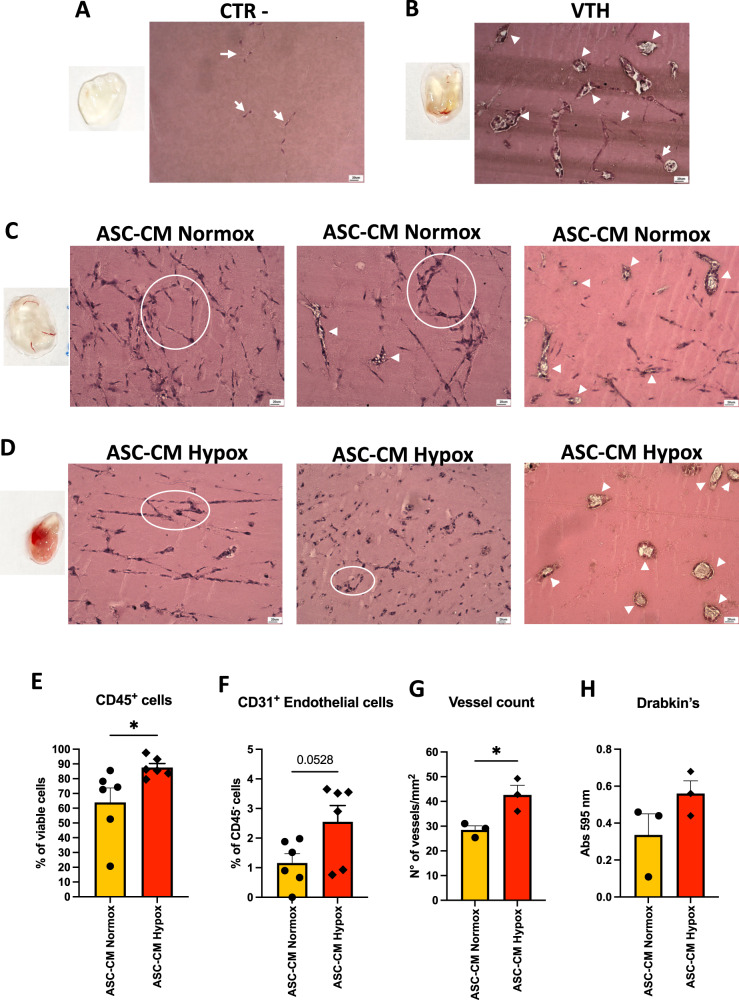
Representative images of macroscopic and optical microscopy observation of Ultimatrix sponge assay. Sponge alone (**A**) or associated with **B** positive control factors (VTH), **C** normoxic ASC- conditioned medium, **D** hypoxic ASC- conditioned medium. Scale bar is 20 μm. **A** Both in the whole scaffold alone and in its histological preparation, the blood vessels are absent, but fibroblasts (arrows) have colonized the scaffold. **B** In the scaffold combined with VTH (positive control), beside the presence of fibroblasts (arrows), mature blood vessels (arrowheads) are noticed. **C**, **D** In the scaffold combined with normoxic ASC- conditioned medium (**C**) or hypoxic ASC- conditioned medium (**D**), the pictures show the formation of vascular-like structures (circles) and the presence of mature blood vessels (arrowheads). Flow cytometry data on CD45^+^ cells (**E**), CD31^+^ endothelial cells (**F**) are showed. **G** Vessel count in the Ultimatrix sponges; **H** hemoglobin evaluation in the Ultimatrix sponges. CTR- (Ultimatrix alone); VTH (VEGF, TNFα, Heparin); ASC-CM Normox (ASC-conditioned media in normoxia); ASC-CM Hypox (ASC-conditioned media in hypoxia). Results are shown as mean ± SEM, *t*-student test, *p < 0.05. FACS results shows values of 6 mice per experimental condition (*n* = 12). Vessel counts and Drabkin’s analysis show values of 3 mice per experimental condition (*n* = 6).

We tested the capability of ASC-CM, collected following 72 h in normoxia or hypoxia, to support angiogenesis, using the in vivo Ultimatrix sponge assay. Visual inspection of collected sponges (Fig. 3A–D) shows the macroscopic presence of blood vessels and blood for those sponges containing VTH (Fig. 3B), ASC-CM in normoxia (CM ASC Normox, Fig. 3C) and ASC-CM in hypoxia (CM ASC Hypox, Fig. 3D).

Histological analysis on the same Ultimatrix sponges confirmed the presence of a vascular network in the sponges with VTH (Fig. 3B), CM ASC Normox (Fig. 3C) and CM ASC Hypox (Fig. 3D), with the formation of more stable vascular-like structures in sponges with the CM ASC Hypox (Fig. 3D).

FACS analysis revealed that the excised Ultimatrix sponges supplemented with the CM ASC Hypox exhibit increased infiltration of CD45^+^ leuckocytes (Fig. 3E), and CD31^+^ endothelial cells (Fig. 3F), compared to those with the CM ASC Normox. Blood vessel count (Fig. 3G) and hemoglobin detection within sponges by Drabkin’s assay (Fig. 3H) confirmed the trend observed by FACS analysis.

Part of the excised Ultimatrix sponges was used for SEM characterization. After 6 days from grafting, representative pictures of SEM indicated that, as already observed in FWM, Ultimatrix alone showed a rearrangement of collagen fibers together with the presence of newly formed fibers synthetized by fibroblasts that have colonized the scaffold. This assumption was reinforced by optical microscopy and by the image at higher magnification (Supplementary Fig. 2).

**Fig. 4:**
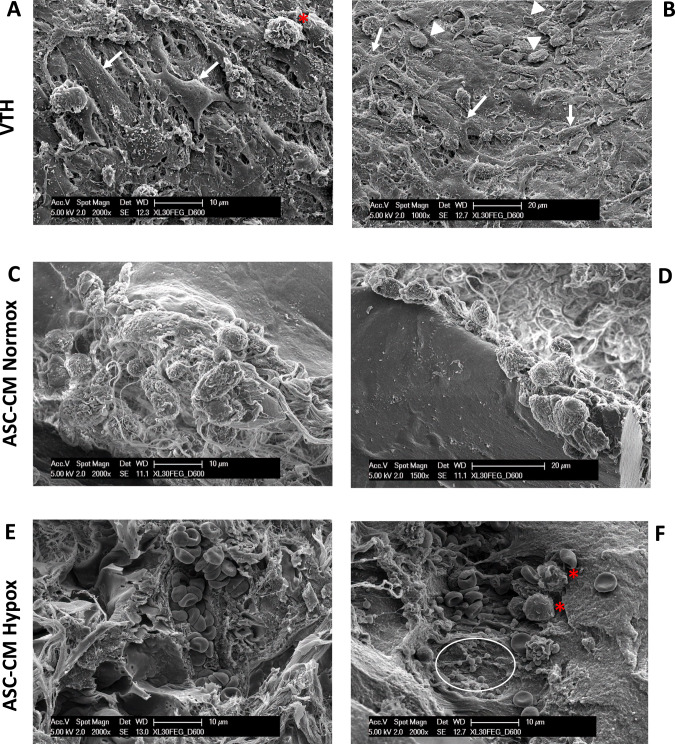
Representative SEM images of the Ultimatrix associated with the designed formulations. **A**, **B** VTH/positive control; **C**, **D** ASC-conditioned media in normoxia (ASC-CM Normox); **E**, **F** ASC-conditioned media in hypoxia (ASC-CM Hypox). Scale bars are indicated in the pictures. In **A**, picture magnification ×2000, and in **B** picture magnification ×1000, the scaffold appears colonized by fibroblasts (arrows), erythrocytes (arrowheads) and a leucocyte (*). In **C**, picture magnification ×2000, numerous cells are interposed among newly formed collagen fibers. In **D**, picture magnification ×1500, endothelial cells are around the cavity of a vessel. In **E**, picture magnification ×2000, mature vessel, full of erythrocytes is evident. In **F**, picture magnification ×2000, leucocytes (*) and platelets (circle) are noticed.

When Ultimatrix was associated with VTH, rather that CM ASC Normox or Hypox (Fig. 4), samples appeared massively colonized by cells including blood cells, clear suggestion of vascularization within the scaffold. In detail, in VTH condition (Fig. 1A), fibroblasts (arrows) and leucocytes, probably macrophages (*), colonized the Ultimatrix, and some erythrocytes were observed as well (Fig. 1B, harrow heads). In CM ASC Normox, (Fig. 4C), numerous cells, interposed among newly formed collagen fibers, were noticed and, interestingly, endothelial cells were detected around the cavity of a vessel (Fig. 4D). Considering that the arrangement of SEM samples barely allows the observation of the complete blood vessel structure, in CM ASC Hypox, we observed a mature vessel full of erythrocytes (Fig. 4E), and infiltration of leucocytes (*) and platelets (circle) (Fig. 4F).

### Characterization of ASC-CM in hypoxia vs normoxia conditions

**Fig. 5:**
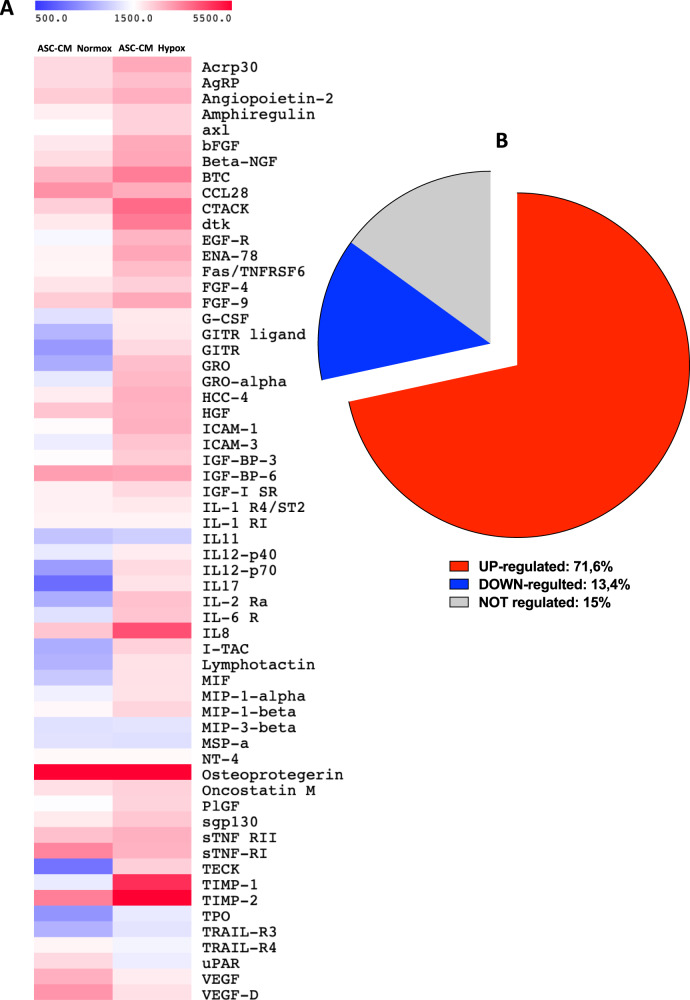
Secretome analysis of conditioned media. **A** Heatmap showing the overall array with all the factors detected in ASC-CMs in normoxic (ASC-CM Normox) and hypoxic (ASC-CM Hypox) conditions. **B** Analysis of the frequency of UP, DOWN and NOT-regulated factors within the overall array. Experiments were performed using CM from two donors, in duplicate (*n* = 4).

Based on the results obtained in the Ultimatrix sponge assay in vivo, we characterized the soluble factors present in ASC conditioned media in normoxia and hypoxia, using a commercially available protein-membrane array (Supplementary Fig. 3). We observed an increase in the secretion of pro-angiogenic factors in CM ASC Hypox, compared to those from the CM ASC Normox (Fig. 5A). Of the 60 pro-angiogenic molecules present in the overall array, we observe the upregulation of 43 factors (71.6%), while only 8 (13.4%) and 9 (15%) were found to be downregulated or non-regulated, respectively, between CM ASC Hypox, and CM ASC Normox (Fig. 5B).

**Fig. 6:**
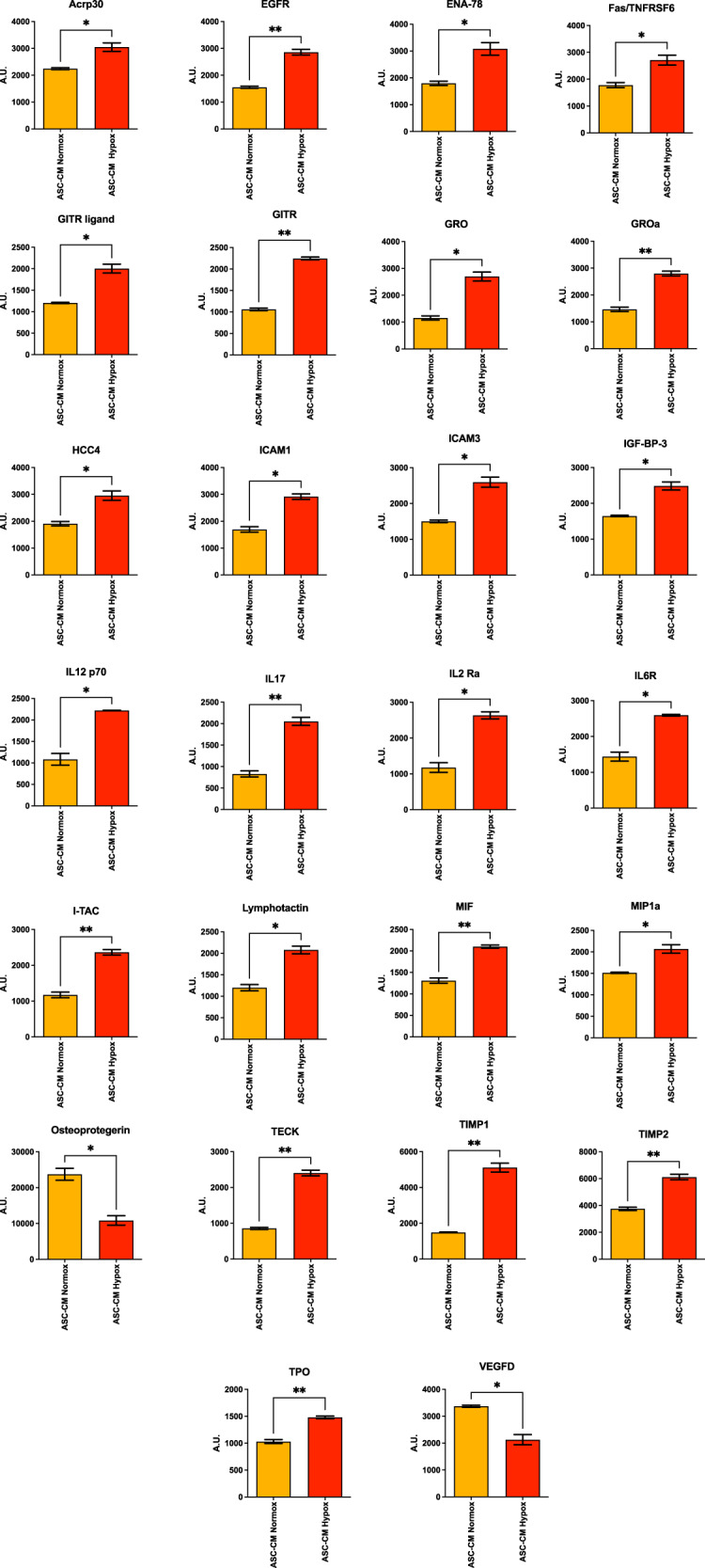
Secretome analysis of conditioned media. Single bar graphs showing the modulation of the soluble factors, by comparing ASC-CMs in normoxic (ASC-CM Normox) and hypoxic (ASC-CM Hypox) conditions. Results are shown as mean ± SEM, *t*-student test, *p < 0.05; **p < 0.01. Experiments were performed using CM from two donors, in duplicate (*n* = 4).

Among the upregulated factors we found molecules involved in different aspects of angiogenesis and endothelial cell activation (Figs. 5, 6, Supplementary Fig. 4) that include: Adiponectin (Acrp30) (Fig. 6A), a molecule linking metabolism and angiogenesis; Epidermal Growth Factor Receptor (sEGFR) (Fig. 6A), involved in angiogenesis; Epithelial-derived Neutrophil-Activating Protein 78 (ENA-78/CXCL5) (Fig. 6A), involved in neutrophils recruitment and activation; Glucocorticoid-induced tumor necrosis factor receptor-related protein/ligand (GTIR-ligand/GITR) (Fig. 6), involved in inflammatory angiogenesis; Growth-regulated proteins alpha, beta and gamma (GROa/b/g or CXCL1/2/3) (Fig. 6), involved in neutrophils and macrophages recruitment and, in particular, GROa (Fig. 6), that exerts autocrine effect on endothelial cells supporting migration and proliferation; macrophage migration factor (MIF) (Fig. 6), involved in inflammatory angiogenesis and macrophage migration; Macrophage inflammatory protein-1α (MIP1α/CCL3) (Fig. 6), involved in cell migration, and chemoattraction of monocytes/macrophages, neutrophils, and mast cells; thrombopoietin (TPO) (Fig. 6), involved in endothelial cell motility; Tissue inhibitor of metalloproteinase 1 and 2 (TIMP1 and TIMP2) (Fig. 6), which are metallopeptidase inhibitors involved in ECM remodeling.

### Effect of ASC-CMs on endothelial cells in vitro

**Fig. 7:**
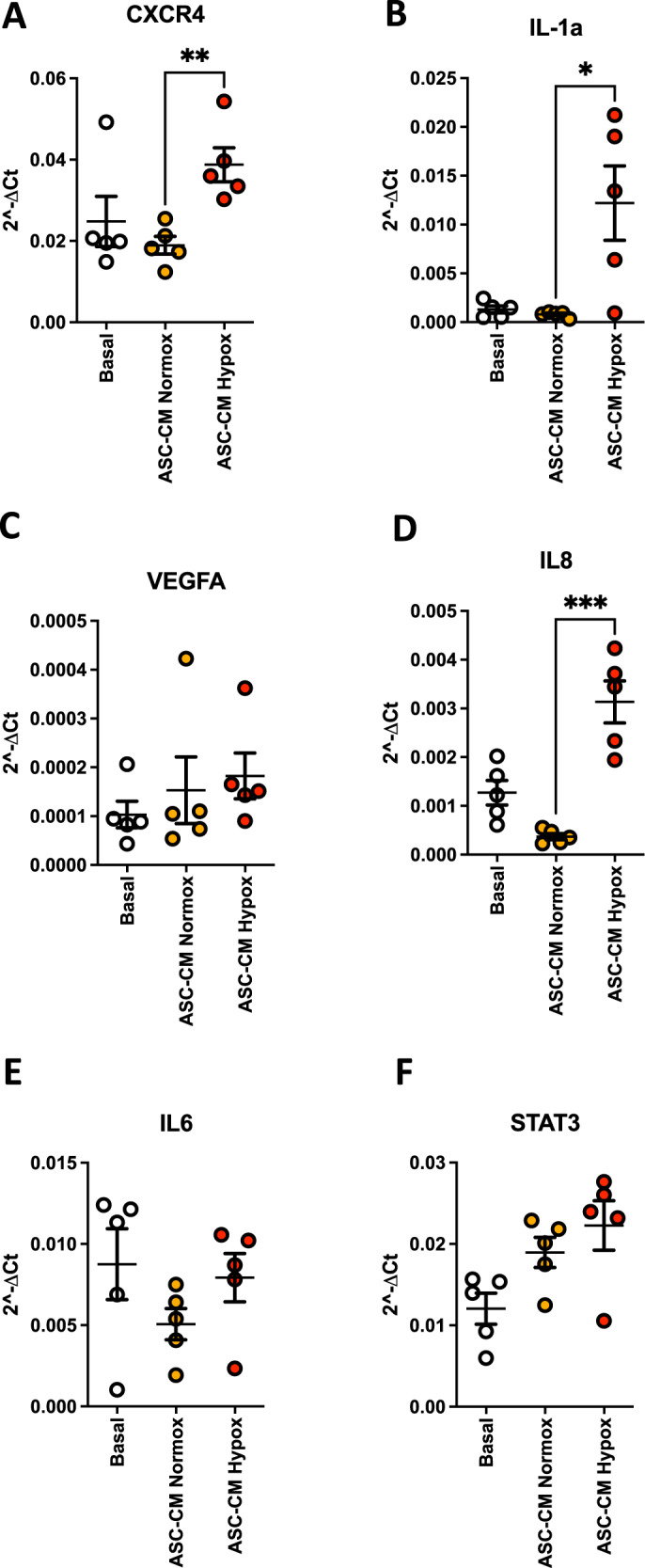
Analysis of pro-angiogenic molecules expressed by endothelial cells exposed to MSC conditioned media. The effects of ASC-CMs in normoxic (ASC-CM Normox) and hypoxic (ASC-CM Hypox) conditions, was assessed by real-time PCR (qPCR), on human umbilical-vein endothelial cells (HUVECs), to detect the transcript levels of CXCR4 (**A**), IL-1α (**Β**), ΙL-1β (**C**), VEGFA (**D**), IL-8 (**E**), IL-6 (**F**), STAT-3 (**G**). Results are shown as mean ± SEM, ANOVA, *p < 0.05; **p < 0.01; ***p < 0.001. Experiments were performed using CM from tree donors, in duplicate (*n* = 6).

Based on the results obtained with the secretome analysis on the presence of pro-angiogenic factors both in normoxic and hypoxic ASC-conditioned media, we tested their ability to induce the expression of pro-angiogenic genes in HUVECs. We found that HUVECs, exposed to hypoxic ASC-CM, increases the expression of C-X-C Chemokine Receptor 4 (CXCR4) (Fig. 7A), Interleukin (IL) 1α (IL-1α) (Fig. 7B) in a statistically significant manner, VEGFA (Fig. 7C) and IL-8/CXCL8 (Fig. 7D), together with a trend of increased expression of IL-6 (Fig. 7E) and Signal Transducer and Activator of Transcription 3 (STAT3) (Fig. 7F) (relevant signaling pathway regulating angiogenesis), compared to HUVECs exposed to normoxic ASC-CM.

## Discussion

Tissue engineering arises as a revolutionary approach in regenerative medicine, offering the necessary knowledge and tools for the development of innovative cues to restore compromised physiological functions of tissues and organs.

Within the best cellular candidate to employ for regenerative medicine approaches MSCs are considered the most effective, being naturally prone to repair tissues in different pathophysiological contexts [17, 32, 45]. As the success of MSC applications in regenerative medicine depends on the appropriate selection of their source, several tissues have been explored. Adipose tissue from adult humans (mammary gland, abdomen, thigh and knee) is easily accessible in large quantities with minimal invasive collecting procedure and provides higher amount of stem cells (500-times more), compared to those that can be obtained by bone marrow [46]. For these reasons, the adipose-derived stem cells (ASCs), a subpopulation of the MSCs, are one of the most popular adult stem cells populations in the field of regenerative medicine [46]. However, recently dental pulp-MSC (DPSCs) are emerging as an attractive source, due to their easy and relatively large accessibility, their higher proliferation rate and maintenance of stemness [45].

This scenario encourages the use of MSCs as a potential tool for regenerative medicine, including cardiovascular regeneration; however, considering the biological and normative restrictions connected with cell therapies, the interests in cell derivatives, such as secretome, has strongly grown in the last decades.

Since revascularization is necessary for efficient tissue regeneration of the damaged area, a remarkable number of strategies, involving the use of MSCs, substrate materials, and biochemical cues emerged. Here, we explored the capability of ASCs and their derivatives (cell-protein extract and conditioned medium), to exert, in vivo, pro-angiogenic activity. SEM analysis revealed similar capabilities of the employed formulations in supporting the generation of blood vessels. These results were confirmed by FACS analysis that showed similar frequency of CD31^+^ endothelial cells infiltrating the scaffold. Interestingly, a similar outcome was found for CD206^+^ scaffold-infiltrating M2-like macrophages. This population identifies a specific polarized macrophage subset with pro-angiogenic properties [12–15]. Together, these results support the hypothesis that ASCs can enhance angiogenesis by directly interacting with endothelial cells, or indirectly, by using M2-like macrophages as bystander cells.

We next validated the in vivo experiments using a different scaffold, the Ultimatrix which resembles the biological ECM milieu. In our model, we explored the contribution of hypoxia, an environmental condition largely known to support angiogenesis. We observed that the pro-angiogenic effects were greater in Ultimatrix sponges associated with ASC-CM obtained in hypoxic conditions. These results are also supported by the secretome arrays that revealed an increase in pro-angiogenic soluble factors in ASC-CM obtained in hypoxia, compared to those from the ASCs grown in normoxia. In hypoxic conditions, 71.6% of upregulated soluble factors are related to different phases of angiogenesis such as: Acrp30/Adiponectin, a molecule linking metabolism and angiogenesis, sEGFR, a cell growth factor involved in angiogenesis, GTIR-ligand/GITR, involved in inflammatory angiogenesis, GRO and GROα, involved in macrophage recruitment, ENA-78/CXCL5, participating to neutrophil recruitment and activation, IL-8/CXCL8, involved in endothelial cell recruitment, MIF, involved in inflammatory angiogenesis and macrophage migration, MIP1α/CCL3, contributing to cell migration, and chemo-attraction of monocytes/macrophages, neutrophils, and mast cells, TPO, involved in endothelial cell motility, TIMP-1 and TIMP-2, involved in ECM remodeling.

We used the same ASC-CMs of the in vivo experiments, to test, in vitro, their capability to induce a pro-angiogenic transcriptional program on HUVEC. We observed increased expression of CXCR4, IL-1α, VEGF, IL-8/CXCL8, IL-6 and STAT3, all genes associated with angiogenesis.

Furthermore, in in vivo experiments, we proved that hypoxia is the optimal culture condition to ensure the production of ASC-CMs rich in pro-angiogenic/tissue repair soluble factors.

A still uninvestigated feature of ASC-CM is its content in extracellular vesicles (EVs). EVs could be another important mediator of the pro-angiogenic effect of ASC-CM and an exhaustive characterization of EVs and EVs content must be carried out to deeper characterize ASC-CM and to investigate novel mechanisms involved in angiogenic promotion.

Finally, our results provide evidence that ASC-CM can be potentially used as a cell-free preparation able to support angiogenesis/revascularization. Considering our data, ASC-CM could be potentially used as preparation for pathological conditions that require revascularization as post-ischemic recovery.

## Materials and methods

### Scaffolds and matrices

Integra® Flowable Wound Matrix (FWM), was kindly provided and characterized by LifeSciences Corporation (Plainsboro, NJ, USA). The Cultrex Ultimatrix (Biotechne) (Minneapolis, MN, USA) at 10 mg/ml of concentration was used as matrix for in vivo angiogenesis matrix sponge assay [47].

### Cell culture and maintenance

Adipose-derived mesenchymal stem cells (ASCs) were isolated from mammary adipose tissue of two healthy women (40 and 57 years old), who underwent breast reduction surgery. The subjects gave their informed consent for inclusion in the study. Subjects were non-smokers, with no history of metabolic disorders, weren’t under treatments at the time of the medical procedure and had not experienced any significant weight loss from dieting (Body Mass Index was <20 kg/m^2^).

The study was approved by the institutional review board ethics committees. Subjects were recruited within a clinical protocol by “University of Insubria” and “Ospedale di Circolo Fondazione Macchi” and approved by the institutional Ethical Committee (protocol no. 30224, April 2013) according to the Helsinki Declaration of 1975 as revised in 2013. The complete characterization of the ASCs has already been published in [48]. ASCs were isolated according to the Gronthos & Zannettino protocol modified in our laboratory [48]. See Supplementary Method section for ASC and HUVEC (ATCC) (Manassas, VA, USA) cell culture.

### Generation of conditioned media (CMs) and protein extract

ASC-CMs were prepared as described in [49]. Briefly, when cells reached 70–80% confluence, media were removed, and cells were washed twice with Phosphate Buffer Saline (PBS). Fetal bovine serum (FBS)-free Dulbecco’s Modified Eagle Medium (DMEM) was added, and cells were incubated for 48–72 h, depending on the scaffold used, in normoxic (21% O_2_) and hypoxic (2% O_2_) conditions. The media were removed and centrifuged at 1000 × *g* for 10 min to avoid contamination of cell debris. To maximize protein contents in the collected CMs, cell media were concentrated using the Amicon Ultra 15 ml Centrifugal filter device (Millipore, Darmstadt, Germany), with a 3 kDa cut-off, following manufacturer’s instructions. Briefly, 13 ml of ASC-CMs were loaded into the tubes and centrifuged at 5000xg for 60 min at 4 °C; the concentrated media were collected and stored at −80 °C until use. Cell protein extracts were prepared by harvesting confluent cells, following a centrifugation step at 1000 × *g*. Cell pellets were suspended in 3 ml of fresh serum-free medium and mechanically lysed. The protein extracts were used immediately after preparation.

### In vivo FWM assay

Seven-week-old male athymic BALB/c nude mice (Crl:CD1-Foxn1nu086) were obtained from Charles River (Calco, Lecco - Italy). The animal studies were approved by the University of Insubria Ethical Committee and by the Italian Ministry of Health, in accordance with the Italian D.Lgs 26/2014. Twelve animals have been used for the experimental procedures. Mice were housed in a conventional animal facility with 12 h light/dark cycles and fed ad libitum. Experiments were performed following the Italian and European Community guidelines (D.L. 2711/92 No.116; 86/609/EEC Directive), the 3 Rs declaration and within an approved protocol by the institutional ethics committee. Grafting procedures were performed as detailed in [23] and Supplementary Method section.

### In vivo Ultimatrix sponge assay

The effects of ASC-CMs (normoxia Vs hypoxia) on the induction of angiogenesis were assessed, in vivo, using the Ultimatrix sponge assay. For Ultimatrix sponge preparation see Supplementary Method section. Seven-week-old male athymic BALB/c nude mice (Crl:CD1-Foxn1nu086, Charles River) were used. Each mouse (6 animals per experimental group) was subcutaneously injected with 600 μl, into the right flank. All animals were housed in a conventional animal facility with 12 h light/dark cycles and fed ad libitum. Experiments were performed in accordance with the Italian and European Community guidelines (D.L. 2711/92 No.116; 86/609/EEC Directive), the 3 Rs declaration and within an approved protocol by the institutional ethics committee. Ultimatrix sponges were excised from mice, 6 days following injections, and used for hemoglobin measurement, histological analysis, and fluorescence activated cell sorting (FACS) analysis.

### Characterization of ASC-conditioned media

The ASC-CMs were analyzed using the Human Cytokine Array C7 (RayBiotec) (Peachtree Corners, GA, USA), according to the manufacturer’s indication and as in [50–52]. For detailed procedures, see Supplementary Method section.

### Flow cytometry

For FWM scaffolds, samples were digested in a type II collagenase solution (3 mg/ml) for one hour at 37 °C under mechanical agitation every 10 min. To obtain a single cell suspension, the digested scaffolds were filtered with a 100 μm pore cell strainer (BD Biosciences) (Franklin Lakes, NJ, USA) and used for flow cytometry analysis.

For Ultimatrix sponges, excised plugs were passed through a 70 μm pore cell strainer (BD Biosciences), using a syringe plugger until complete dispersion, then used for flow cytometry analysis.

The single cell suspension was stained with Fixable/Viable die (BD Biosciences) and the following combinations of anti-mouse monoclonal antibodies (all from BD Biosciences): FITC-CD31, BUV-395-CD45, PE-CF596-F4/80, BV-421-CD80, Alexa Fluor-647-CD206, (for Integra® FWM scaffold), FITC-CD31, BUV-395-CD45 (for Ultimatrix sponges). Following staining, cells were washed in PBS and analyzed using a FACS Fortessa x20 (BD Biosciences), equipped with 5 lasers. Viable cells were identified based on doublet exclusion (side scatter area/SSC-A Vs side scatter height/SSC-H), morphology (forward scatter area/FSC-A Vs side scatter area/ SSC-A) and as negative cells for the Fixable/Viable die. Viable cells were used to identify different cell types as follows: CD45^-^ cells (stromal cells), CD45^-^CD31^+^ cells (endothelial cells), CD45^+^ cells (total leukocytes), CD45^+^F4/80^+^ cells (total macrophages), CD45^+^F4/80^+^CD80^+^ cells (M1-like macrophages), CD45^+^F4/80^+^CD206^+^ (M2-like macrophages). Due to the limited cell numbers in Ultimatrix sponges, only CD45^+^ and CD45^-^CD31^+^ cells were detected with acceptable fluorescence signals. FACS data were acquired with the FACS Diva software (BD Biosciences), then analyzed with the FlowJo v10 software (BD Biosciences).

### Real-time PCR

HUVEC (5 × 10^5^ cells in 100 mm cell culture dish) were treated with 50 μg of total protein of ASC-CMs, obtained under normoxic and hypoxic conditions, in serum-free media, for 6 h. Cells were collected in trizol reagent and stored at −80 °C until use. Total RNA was extracted using the small RNA miRNeasy Mini Kit (Thermo Fisher) and quantified by Nanodrop Spectrophotometer. 500 ng of total RNA was reverse transcribed using SuperScript VILO cDNA synthesis kit (Thermo Fisher Scientific, USA). Real-time PCR was performed using SYBR Green Master Mix (Thermo Fisher) on QuantStudio 6 Flex Real-Time PCR System Software (Thermo Fisher). The 18S RNA gene was used as housekeeping, and HUVECs with their serum-free media were used as baseline control. Primer sequences are provided in Supplemental Table 1.

### Scanning electron microscopy (SEM) analysis

Samples were fixed in 1% Karnowsky solution in 0.1 M sodium cacodylate buffer (pH 7.4) overnight at 4 °C and preserved in the same buffer. The specimens were dehydrated in graded ethanol and dried in hexamethyldisilazane (Sigma Aldrich, Milano, Italy), mounted on aluminum stubs, then covered with 10 nm of gold (Emitech K550) and observed with a Philips SEM-FEG XL-30 (Eindhoven, The Netherlands).

### Statistical analysis

Statistical analysis was performed using the GraphPad Prism software v9 (GraphPad Prism Inc., San Diego, CA, USA), with Student *t*-test or one-way ANOVA, followed by Tukey’s post-hoc test correction. Results are shown as mean ± SEM. *P* values ≤ 0.05 were considered statistically significant.

## Supplementary information

SUPPLEMENTARY METHODS AND SUPPLEMENTARY FIGURE LEGENDS

Supplementary Figure 1

Supplementary Figure 2

Supplementary Figure 3

Supplementary Figure 4

## Data Availability

All data generated or analyzed during this study are available from the corresponding author on reasonable request.
