# Supplementary material for: Adipose mesenchymal stem cell-derived soluble factors, produced under hypoxic condition, efficiently support in vivo angiogenesis
Source: Cell Death Discov. 2023 May 23;9:174. doi: 10.1038/s41420-023-01464-4 (PMC10205717; doi:10.1038/s41420-023-01464-4)
Supplement: Supplementary file 1 — SUPPLEMENTARY METHODS AND SUPPLEMENTARY FIGURE LEGENDS [file 41420_2023_1464_MOESM1_ESM.docx]

**SUPPLEMENTARY METHODS AND SUPPLEMENTARY FIGURE LEGENDS**

**SUPPLEMENTARY METHODS**

**Scaffolds and matrices**

Integra® Flowable Wound Matrix (FWM) is a 3-D porous biocompatible matrix comprised of granulated cross-linked bovine tendon collagen and glycosaminoglycan. Quantitative measurements revealed that the fibers exhibited a 2 μm thickness and 120 μm length with an average inter-fiber distance of 45 μm. It is commonly used for the treatment of tunneled and irregular wounds, which are often associated with excessive scar tissue formation. The device, supplied freeze-dried, can be hydrated with culture media. In this way, the scaffold acquires a gel-like consistency making it optimal for subcutaneous injection. More information about the structure, porosity and biocompatibility is available at the Integra web site (Integra.www.integralife.com).

**Cell culture and maintenance**

Briefly, the stromal vascular fraction (SVF) was obtained by collagenase type II digestion (Sigma Aldrich, Milano - Italy) at 37°C, for 1 h, under agitation. SVF was filtered with a 100 μm cell strainer (BD Bioscineces) and centrifuged at 180xg for 10 min (Eppendorf 5804R, Milan, Italy); the resulting pellet was washed with erythrocyte lysis buffer (154 mM NH4Cl, 10 mM KHCO3, and 1 mM EDTA), then cells were seeded in T25 flasks and maintained at 37°C, 5% CO_2_. Following 24 h, non-attached cells were removed. Cells were grown in DMEM:DMEM F12 1:1 (Sigma Aldrich, Milano - Italy), supplemented with 2 mM L-Gln, 1% penicillin-streptomycin, 0.1% gentamicin, and 10% FBS. Due to the abundant number of ASCs necessary to run all the experiments and given that from P3 to P5 are considered as early passages expressing the same secretome, cells were subsequently cultured in T75 flasks and used at P5, for all experiments.

Human Umbilical-Vein Endothelial Cells (HUVECs) were purchased by ATCC and cultured in endothelial cell basal medium (EBM, Lonza), supplemented with endothelial cell growth medium SingleQuots and Growth Factors (Lonza), 2 mM l-glutamine (Euroclone), 100 U/ml penicillin, and 100 μg/ml streptomycin (Euroclone), and maintained at 37°C, 5% CO_2_. HUVECs were used between passages 3-4. All cell types were regularly tested for the absence of mycoplasma contamination.

***In vivo* Integra® Flowable Wound Matrix**

The grafting was carried out following isoflurane anesthesia. A 0.5 cm incision was created in mice’s backside to inject the scaffolds between muscle and subcutaneous layer using a syringe with a luer-lock connector and a flexible injector. The grafts consisted of: 1) Integra® FWM hydrated with fresh FBS-free culture medium; 2) Integra® FWM hydrated with fresh culture medium containing 3×10^6^ MSCs; 3) Integra® FWM hydrated with fresh culture medium containing crude protein extract derived from 3×10^6^ ASCs; 4) Integra® FWM hydrated with ASC-CM derived from 3×10^6^ cells. Each syringe contained a total volume of 3 ml of each formulation and a volume of 200 μl, for each preparation, was injected in any single animal. Incisions were then stitched using surgical sterile strips.

***In vivo* Ultimatrix sponge assay**

Thawed and liquid Ultimatrix (concentration 10mg/mL, Biotechne) was mixed with 50 μg of concentrated CMs from normoxic or hypoxic cultured ASCs, to generate a liquid sponge with a final volume of 600 μL. Negative and positive control mixtures included Ultimatrix (Biotechne) alone or Ultimatrix supplemented with VHT (100 ng/ml VEGF-A, 2 ng/ml TNF-α and 25 U/ml heparin) respectively.

**Detection of hemoglobin by colorimetric assay**

200 μl of PBS from mechanically processed Ultimatrix sponges were mixed with 800 μL of liquid Drabkin’s reagent. The mix was incubated at room temperature for 20 minutes, under gentle shaking. Following 20 minutes of incubation, 100 μL per sample were transferred into a 96-well plate and used to read absorbance, at 595 nm, using Spectramax plate reader.

**Optical microscopy and vessel counting**

The inner portions of the scaffolds were fixed in 4% paraformaldehyde solution, dehydrated with ethanol (70%, 90%, 95%, 100%), embedded in paraffin and sectioned using an RMC-RM3 rotary microtome (TiEsseLab, Milan - Italy). Five non-consecutive sections (6 μm) per sample were mounted on glass slides, stained with H&E solution, following classical procedures, and analyzed using ISCapture software to evaluate the number of vessels/mm^2^ in each condition.

**Characterization of ASC-conditioned media by protein array**

50 μg of ASC-CM from normoxic or hypoxic conditions were used. Signal intensity, as chemiluminescent dots, was detected using the Alliance Q9 instrument (UVITEC). Arrays were computed scanned using the Alliance Q9 instrument (UVITEC), and optical density, for each dot/spot, was determined using the ImageJ software and Dot plot Analyzer plugin.

**SUPPLEMENTARY TABLE1**

| **Primers target** | **Forward** | **Reverse** |
| --- | --- | --- |
| hCXCR4 | ATCAGTCTGGACCGCTACCT | CCACCTTTTCAGCCAACAGC |
| hIL-1a | TGTATGTGACTGCCCAAGATGAAG | AGAGGAGGTTGGTCTCACTACC |
| hIL-6 | GAGGAACAAGCCAGAGCTG | GGTCAGGGGTGGTTATTGC |
| hIL-8 | CCTGATTTCTGCCAGCTCTGTG | GTGGTCCACTCTCAATCACTCTC |
| hVEGFA | TTGCCTTGCTGCTCTACCTCCA | GATGGCAGTAGCTGCGCTGATA |
| hSTAT-3 | CTTTGAGACCGAGGTGTATCACC | GGTCAGCATGTTGTACCACAGG |

**SUPPLEMENTARY FIGURE LEGENDS**

**Supplementary Fig. 1 Representative images of SEM observation of the scaffold FWM after grafting.** Picture shows that in the FWM alone (A), collagen ultrastructure was maintained. Magnification 500x. Picture of FWM associated with ASCs (B) evidences a scaffold reorganization in which newly formed collagen fibrils are present. Magnification 5000x. Detail at higher magnification (C), 20000x, of the picture B. Scale bars are indicated in the pictures.

**Supplementary Fig. 2 SEM observation of the Ultimatrix sponge after grafting**. Picture shows that in the Ultimatrix alone, the ultrastructure was maintained (A). Magnification 2500x. Picture of Ultimatrix associated with ASC-CM Hypox (B) evidences a scaffold reorganization in which newly formed collagen fibrils are present. Magnification 20000x. Scale bars are indicated in the pictures.

**Supplementary Fig. 3 Secretome analysis of conditioned media.** The overall array and related spots are showed for ASC-CMs in normoxic (A) and hypoxic (B) condition. The plot matrix for the used array and related factors detected is showed in (C). Experiments were performed using CM from two donors, in duplicate (n=4).

**Supplementary Fig. 4 Secretome analysis of conditioned media.** Single bar graphs showing the modulation of the soluble factors, by comparing ASC-CMs in normoxic (ASC-CM Normox) and hypoxic (ASC-CM Hypox) conditions. Results are shown as mean ± SEM, t-student test. Experiments were performed using CM from two donors, in duplicate (n=4).
